# Inflammation and Oxidative Stress as Common Mechanisms of Pulmonary, Autonomic and Musculoskeletal Dysfunction after Spinal Cord Injury

**DOI:** 10.3390/biology11040550

**Published:** 2022-04-01

**Authors:** Cristián Rosales-Antequera, Ginés Viscor, Oscar F. Araneda

**Affiliations:** 1 Physical Medicine and Rehabilitation Unit, Clínica Universidad de los Andes, Santiago 8320000, Chile; carosales@miuandes.cl; 2 Integrative Laboratory of Biomechanics and Physiology of Effort, LIBFE, School of Kinesiology, Faculty of Medicine, Universidad de los Andes, Santiago 8320000, Chile; 3 Physiology Section, Department of Cell Biology, Physiology and Immunology, Faculty of Biology, Universitat de Barcelona, 08028 Barcelona, Spain; gviscor@ub.edu

**Keywords:** spinal cord injury, pathophysiology, inflammation, oxidative stress

## Abstract

**Simple Summary:**

When a spinal cord injury occurs, the neurons that regulate our voluntary movements, those involved in environment and somatic perception and those that regulate vegetative functions are affected. Once neuronal damage is established, the cells of other tissues are also affected in their functions, altering the interaction between organs and altering the proper functioning of the organism. Multiple studies in animal models, as well as in humans, have recognized as factors involved in organ damage the imbalance between the formation of highly reactive molecules called pro-oxidants and defensive mechanisms called antioxidants. Closely associated with this phenomenon, the inflammatory response is also pathologically activated. In this narrative review, we have analyzed the information involving these pathological processes at the level of the lung, the autonomic nervous system and the skeletal musculature after spinal cord injury. Knowing the abnormal functioning mechanisms that occur after a spinal cord injury not only offers a better understanding of the organic events but also offers future possibilities for therapeutic interventions that may benefit the thousands of patients suffering this pathology.

**Abstract:**

One of the etiopathogenic factors frequently associated with generalized organ damage after spinal cord injury corresponds to the imbalance of the redox state and inflammation, particularly of the respiratory, autonomic and musculoskeletal systems. Our goal in this review was to gain a better understanding of this phenomenon by reviewing both animal and human studies. At the respiratory level, the presence of tissue damage is notable in situations that require increased ventilation due to lower thoracic distensibility and alveolar inflammation caused by higher levels of leptin as a result of increased fatty tissue. Increased airway reactivity, due to loss of sympathetic innervation, and levels of nitric oxide in exhaled air that are similar to those seen in asthmatic patients have also been reported. In addition, the loss of autonomic control efficiency leads to an uncontrolled release of catecholamines and glucocorticoids that induce immunosuppression, as well as a predisposition to autoimmune reactions. Simultaneously, blood pressure regulation is altered with vascular damage and atherogenesis associated with oxidative damage. At the muscular level, chronically elevated levels of prooxidants and lipoperoxidation associated with myofibrillar atrophy are described, with no reduction or reversibility of this process through antioxidant supplementation.

## 1. Introduction

Globally, the WHO estimates that between 250,000 and 500,000 people suffer from a spinal cord injury (SCI) on an annual basis, mainly associated with traffic accidents, falls or acts of violence [1].

Once the traumatic event is identified using traction and compression, ischemia-hypoxia in the areas of vascular compromise is shown involving the release of pro-inflammatory cytokines [2] and the attraction of immune cells that damage local nervous tissue via necrosis/apoptosis, both directly and through the promotion of autoimmune-type phenomena [3]. Consequently, the degree of impact on organic functions will depend on the level of the spinal cord injury. The compromise of spinal cord function has both acute and chronic effects that simultaneously alter voluntary motor control and, thus, the action of the functional bone/musculoskeletal dyad of the locomotor system, as well as the various functions of the autonomic nervous system, which have an impact on the genito-urinary control, the motility of the digestive system, the control of the respiratory system and the action of the cardiovascular system [4]. In the long term, these events promote changes in body composition [5] and increase the development of infectious [6], autoimmune processes [7], disorders of endocrine function and metabolic control that are associated with increased morbidity and mortality [8].

Secondary to the SCI, patients show alterations in the functions of different body systems. Thus, at the respiratory level, the functional residual capacity (FRC) and the forced expiratory volume in one second (FEV1) decrease, the respiratory pattern changes and the maximum inspiratory/expiratory pressure decreases [9]. Furthermore, this group has the mechanism of coughing altered by a stiffer thoracic cavity that generates greater respiratory work along with the presence of bronchial obstruction and hyperresponsiveness [10]. In addition, there are respiratory disorders during sleep, such as obstructive sleep apnea, which causes chronic intermittent hypoxia [9,11]. These effects can explain the high prevalence of symptoms like dyspnea, chronic cough, bronchial hypersecretion and an increased susceptibility to respiratory tract infections [9,12].

Another affected system in patients with SCI is the autonomic nervous system, which alters cardiovascular and respiratory regulation, leading to variable and abnormal functioning [1]. This autonomous deregulation induces neuroendocrine changes that can trigger the imbalance of the redox state and promote chronic tissue inflammation [13]. The alteration of the functioning of the autonomic nerve pathways generates a loss of efficiency in the control of the cardiovascular system, affecting both the regulation of blood pressure and heart rate [14]. It also affects the body’s cooling mechanisms, apparently by the alteration of the vasomotor tone and a decrease in afferents towards the thermoregulatory centers, which generates a loss of the ability to sweat [15]. Finally, the deregulation of this system has also been associated with a case of immune deficiency [16].

Skeletal muscle is also affected prematurely after spinal cord injury, rapidly suffering significant atrophy [17]. Regarding the mechanisms involved in this process, it has been possible to identify the participation of oxidative imbalance as a key factor involved in the phenomena of muscle protein synthesis and degradation [18,19,20].

There is currently abundant information that involves the damage mechanisms associated with SCI, inflammation and oxidative stress. In this way, we have set ourselves the objective of describing, through a narrative review, the scientific evidence obtained from animal models and studies carried out in humans that involve these pathogenic factors with damage to the respiratory, autonomic and muscular systems typical of SCI. The selection of the chosen works was limited to original works and of review articles accessible through the PubMed search engine without publication date limits.

## 2. Respiratory System

Ventilatory changes: Spinal cord injury involves nerve damage that is generally described from the sensory to the muscular system. However, the muscle paralysis that the spinal cord injury generates not only involves the locomotor musculature, but rather, depending on the level of the injury, it also affects the respiratory musculature to a greater or lesser extent, which can establish changes in ventilatory pattern [21].

The respiratory musculature generates the alveolar pressure changes necessary to produce pulmonary ventilation. Thus, its involvement can lead to poor performance of the cough mechanism and atelectasis caused by deficient pulmonary ventilation [21,22].

Mateus et al. [23], through spirometry and maximal static respiratory pressure tests, assessed the muscle strength involved in forced pulmonary ventilation and its relationship with the ability to generate effective coughs in spinal cord injured patients, proving that the vital capacity correlates directly with the effectiveness of the cough, while maximal inspiratory pressure was closely related to having a higher vital capacity. It was also described with a greater involvement at higher lesion levels (C4-C5), while the tests were practically normal on patients who suffered paralysis in lower limbs (T7-L3). Therefore, the intercostal muscles, weakened, lose the possibility of reaching full lung capacity. In this way, disuse of the respiratory intercostal musculature is gradually produced, generating muscular fibrosis and a decrease of thoracic distensibility [24]. Malas et al. [25] assessed the diaphragmatic changes through ultrasonography, observing that despite the lower results obtained in the pulmonary function tests, the diaphragmatic thickness was greater in this group, which was interpreted as overcompensation, due to a greater load and respiratory rate in this muscle. The respiratory muscular weakness in spinal cord injured patients has been confirmed over the years, so that even the acceptability criteria of some functional tests such as spirometry have had to be specifically adapted to this population [26]. In fact, as early as 1980, doubts had already arisen regarding the implications and changes in the respiratory system that muscle weakness could cause. In this year, Forner et al. [27] assessed the effect of respiratory musculature paralysis on pulmonary volume, describing a drop in vital capacity and expiratory reserve volume, in addition to a decrease in peak expiratory flow. Likewise, Anke et al. [28] assessed the pulmonary function of 56 tetraplegic patients, with over six months post spinal cord injury finding a decrease in the volume of expiratory reserve and vital capacity. Baydur et al. [29] described that the forced expiratory volume, the FEV1 and the inspiratory capacity increased as the lesion was lower, reinforcing the conclusions of the previously described studies. In addition to these findings, it was observed that, in the supine position, unlike what has been described in healthy subjects, inspiratory capacity increased, which may be attributable to the compression generated by gravity in the abdominal wall, decreasing the greater distensibility of the abdomen that subjects with SCI have, thus giving them better leverage points for the action of the diaphragm. In the same line of research, Terson de Paleville et al. [30] furthered the study of the effect of the position on pulmonary volumes, describing that when the spinal cord injury was complete, the supine position benefited the forced vital capacity, while the spirometric values decreased in this position in subjects with an incomplete spinal cord injury, reinforcing the idea that the supine position on subjects with a high injury may confer some degree of mechanic muscular advantage [30].

From a different point of view, a study was conducted on the predisposing factors of the progressive loss of pulmonary function of spinal cord injured patients. Stolzmann et al. [31] evaluated 174 spinal cord injured patients, who filled out a questionnaire of respiratory health and had a follow-up with serial spirometric assessments over the course of seven years on average. This study showed that the progressive decrease of FEV1 and the FVC was linked to smoking habits, as well as other modifiable factors, more than with the gravity and the level of the spinal cord injury. In a retrospective study, the behavior of the pulmonary function of 173 spinal cord injured patients who were subjected to spirometry controls over time was described. No change in pulmonary function was observed over an average of 23 years, except for a subgroup with a higher body mass index (BMI) who decreased pulmonary volumes over time [32]. This finding is of great significance since a large percentage of spinal cord injured patients will have a forced decrease of energy consumption, which can lead to an increase in fatty tissue, followed by a decrease of muscular tissue, which leads these subjects to a chronic systematic inflammatory state, produced by the release of adipokines, like leptin [33,34,35]. Leptin has action at the pulmonary level, since there are receptors for this adipokine in the alveolar epithelium and in the smooth muscles of the airway [35,36,37]. Just as leptin has a proinflammatory role, adiponectin is also released by the fatty tissue and has an anti-inflammatory role. For this reason, Garshick et al. [35] studied the link between leptin and adiponectin with pulmonary function in spinal cord injured patients. This group of researchers found an inverse relationship between plasmatic leptin levels and FEV1 and FVC values, which might suggest that high leptin levels may also be a factor that influences the decrease in pulmonary function, specifically its vital capacity [35]. These results, which indicate an effect of chronic inflammation in spinal cord injured patients, are consistent with a previous study by the same group, in which an inverse relationship between systemic inflammation and the deterioration of pulmonary function in spinal cord injured patients had been described [38]. The decrease in vital capacity, and in nearly all inspiratory volumes, in spinal cord injured patients, coupled with the loss of thoracic cavity distensibility, is what has led to the description of a chronic restrictive ventilatory pattern in subjects with SCI that predisposes them to generate pulmonary atelectasis [21,22,23,37,39]. Chronic pulmonary restriction could be a problem for these subjects when they are exposed to conditions that force them to increase ventilatory capacity. From intensive care medicine, due to the possible impact of mechanical ventilation, the effect of ventilating restrictive lungs with high flow volumes has been extensively studied, since they generate an inflammatory response, either by damage through alveolar opening and closing (atelectrauma), or by overdistension (volutrauma) [40,41,42]. This injury is caused by the activation of alveolar macrophages due to the expression of damage associated molecular patterns (DAMPs), released by the destroyed cells of the alveolar epithelium, and such activation of the macrophages can induce further injury. In this way, an initial mechanical damage caused by ventilation with high flow volumes would stimulate local inflammatory activation, causing even more damage [43]. If we extrapolate this problem, which is pressing in the context of patients undergoing mechanical ventilation, spinal cord injured patients who have lower pulmonary distensibility and a restrictive ventilatory pattern, we could think that physical exercise, which demands an increase in pulmonary ventilation, could also induce greater inflammation and damage.

West et al. [39] observed changes in cardiopulmonary function in spinal cord injured athletes at rest and described that they maintain a restrictive pattern at rest compared to healthy athletes. Despite this, pulmonary function improves when compared to non-athletic spinal cord injured patients. The fact that persons with SCI, who participate in physical training programs, maintain restrictive ventilatory patterns reinforces the hypothesis that, during physical activity, they would be exposed to greater lung damage than healthy subjects due to the hyperventilation required by exercise. This makes it interesting to evaluate local inflammatory markers at the pulmonary level in persons with SCI during physical activity and compare them with healthy persons.

Bronchial obstruction and hyperresponsiveness: Spinal cord injured persons suffer various respiratory complications from an acute stage of injury, ranging from an increased predisposition to upper respiratory tract infections, to atelectasis and bronchospasm [44]. Chronically, persons with SCI are more exposed to respiratory disorders due to a deficit in mucociliary clearance, decreased ability to exert high expiratory flows, the repeated use of antibiotics that can change the microbiota of the respiratory tract and the generation of disorders in the autonomic nervous system, which can lead to changes in the structure and quantity of mucus, often resulting in recurrent bronchitis [45].

Almendoff et al. [46] evaluated whether there is indeed an obstructive component in spinal cord injured patients, since tetraplegic patients would have partially inhibited sympathetic innervation, being able to maintain parasympathetic innervation, promoting a basal bronchoconstrictor tone. It was observed that 48% of tetraplegic subjects obtained an improvement in both FVC and FEV1 following the use of ipratropium bromide. Later, De Luca et al. [47] added evidence that subjects with SCI, when faced with the use of beta 2 agonist inhalers, also generated an improvement in lung function, reinforcing the idea that these subjects maintain a basal obstructive component. Subsequently, Schilero et al. [10], comparing the response to ipratropium bromide between a tetraplegic group and a paraplegic group, reported that subjects with tetraplegia had a smaller basal airway caliber and a greater response to the use of this bronchodilator. These results reinforce the idea that, in high spinal cord injury, there may be an expression of the parasympathetic system without an opposing effect of the sympathetic system. Schilero et al. [48] compared the effect between albuterol and ipratropium bromide, and observed that both drugs generate a decrease in airway resistance. However, the change in resistance was more marked with ipratropium bromide than with albuterol (71% vs. 47%). These results suggest that vagal tone is the main determinant of the basal increase in bronchoconstriction.

The response to bronchodilators that has been observed in subjects with SCI may also indicate bronchial hyperresponsiveness. Triggers of bronchial hyperresponsiveness have been described, the main ones being histamine release, or some indirect external factors such as the cold and exercise [49,50]. In a study to evaluate bronchial hyperresponsiveness by Dicpinigaitis et al. [51], a group of subjects with cervical spinal cord injury were exposed to a methacholine challenge test, which demonstrated some degree of bronchial hyperresponsiveness in all participants, and furthermore, it was observed that ipratropium bromide was able to completely reverse the effect. The investigators postulated the bronchial hyperresponsiveness observed in this population most likely reflects the loss of sympathetic airway innervation and resultant unopposed cholinergic bronchoconstrictor tone which results from transection of the cervical spine. Blockade of methacholine hyperresponsiveness with ipratropium bromide suggests a muscarinic receptor-mediated phenomenon [51]. Singas et al. [52] conducted another study to see whether bronchial hyperresponsiveness could be motivated by the persons’ previous or current smoking habit. For this purpose, subjects with spinal cord injury, both ex-smokers and non-smokers, were added to the evaluation and underwent a methacholine challenge test, which showed the same results in both groups. The authors thus added a new question, which was to investigate the impact that the decrease in lung volumes generates on the loss of the capacity to maintain a larger airway caliber.

The mechanisms that generate bronchial hyperresponsiveness are diverse, and recently it was described that airway inflammation may play a key role, although the acute mechanisms concerning how inflammation is linked to smooth muscle hyperresponsiveness are still unclear. However, it seems clear that, in chronic inflammation processes, airway remodeling is generated, promoting bronchial obstruction [49]. On the other hand, subjects with SCI, have characteristics that promote systemic inflammation due to their metabolic changes, propensity to be overweight and decreased muscle tissue, which may cause reduced lung function [38]. In addition, there is evidence that these characteristics are associated with an increase of IL-6 and c-reactive protein (CRP) in the blood plasma of spinal cord injured patients, since the levels of these inflammatory markers would have an inverse relationship with lung function [53]. In addition to this, it is relevant to mention the effects that inflammation causes directly in the respiratory system, such as a decrease in mucociliary clearance, changes in microbiota, increased incidence of upper respiratory tract infections and microaspirations [45,54].

**Figure 1:**
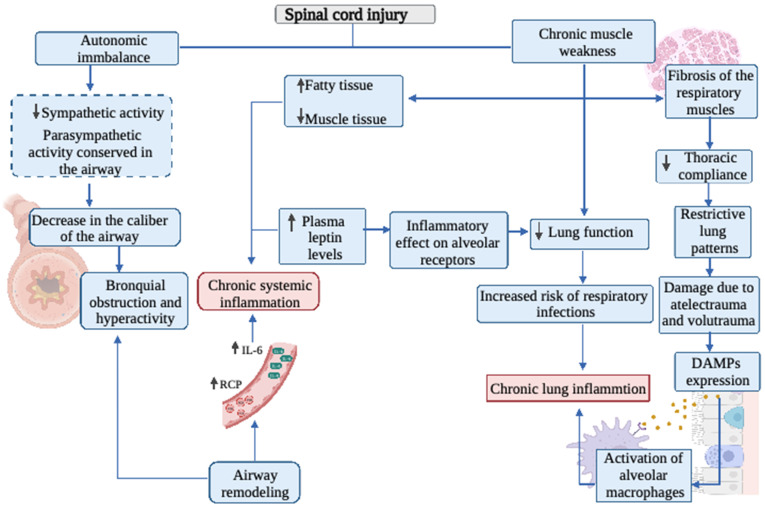
Overview of the role of inflammation and oxidative stress on the respiratory system in spinal cord injured patients. IL-6: Interleukin-6; CRP: C-reactive protein; DAMPs: Damage-associated molecular patterns.

To measure inflammatory markers in the airway, Radulovic et al. [55] used a noninvasive technique, assessing exhaled nitric oxide, and observed a significant increase in nitric oxide levels in tetraplegics compared to the control group, and similar to the group of asthmatic persons. While these findings are not conclusive and require further investigation, they provide indications of the presence of some local inflammatory component in the airway that may be similar to that of asthmatic persons. A summary of the topics discussed in this chapter is presented in Figure 1.

## 3. Autonomic Nervous System

Among the neurological consequences of SCI, there is not only a serious functional compromise of the somatic nervous system, but also of the autonomic nervous system. The sympathetic nervous system, whose activity is affected when the lesion interrupts supraspinal regulatory signals, could generate problems in gastrointestinal motility, urinary continence and hemodynamic control [56,57]. The most severe expression of autonomic system disorder in subjects with SCI is autonomic dysreflexia (AD), which has its origin in the activation of dysregulated sympathetic reflexes, which can generate an exaggerated or deficient response in various functional systems, accompanied by an increase in parasympathetic tone [58]. AD can cause problems, for example, at the cardiovascular level ranging from orthostatic hypotension to severe hypertensive crises [14,54,58]. The incidence of hypertension in high spinal cord injuries is estimated to be around 46%. This incidence of hypertension is also thought to be due to increased vascular stiffness in persons with SCI [54]. The imbalance of physiological functions and loss of homeostasis caused by multiple factors, including AD, produces systemic stress that can lead to a chronic inflammatory state, which manifests as increased cardiometabolic disease and abnormal immune responses, ranging from autoimmune processes to immunosuppression [59].

Severe hypertensive conditions attributable to AD are related to the elevated and dysregulated release of catecholamines, which in the medium to long term generates endothelial damage and promotes atherogenesis, among other vascular pathologies [59,60,61,62]. Noller et al. [59] point out that the management of chronic inflammation in persons with SCI is very relevant to improve their quality of life, and for that it is necessary to understand in a deeper way the role of the autonomic nervous system. Hoekstra et al. [63] studied the systemic inflammatory response to exercise in subjects with SCI. Subjects with a non-cervical injury generated increased catecholamine release and concurrently increased plasma IL-6 after competing in a wheelchair half-marathon event. While a direct relationship was found between the levels of catecholamines released and IL-6 levels, it is not certain that this is due to AD, since AD is more prevalent in subjects with cervical SCI. Sudden changes in arterial pressure and turbulent flows generated in the bifurcation zones of the arteries generate stress in the vascular wall. This promotes increased permeability in these areas to lipoproteins circulating in the blood [64,65]. LDL molecules penetrate such areas more easily into the vessel wall, accumulate in the tunica intima and are subsequently oxidized (oxLDL). These oxidized molecules act as patterns associated with molecular damage (DAMPs), generate endothelial damage and activate proinflammatory processes through pattern recognition receptors (PRRs) [64,66]. Damaged and activated endothelial cells express adhesion molecules and cytokines such as E-selectin, P-selectin, VCAM-1 and ICAM-1, which attract monocytes to the site of the atherosclerotic lesion and promote their conversion to macrophages [67,68]. These activated cells phagocytize cholesterol esters and form foam cells, stimulating an inflammatory reaction with migration of vascular smooth muscle cells that stabilize the atheroma plaque with fibrotic tissue. Simultaneously to the innate inflammatory response, an adaptive response involving different types of T and B cells is triggered against the atheroma plaque and renders it unstable [64,68]. On the other hand, oxidative stress plays a fundamental role in the atherosclerotic process. Thus, the vascular wall has oxidative systems such as xanthine oxidase, mitochondrial respiratory chain enzymes, lipoxygenases, NOX and antioxidant systems, including superoxide dismutase (SOD), catalase and glutathione peroxidase [64,69,70]. Activation of LOX-1, a macrophage receptor that is triggered by oxLDL, induces endothelial oxidative stress by increasing NOX activity which increases hydrogen peroxide and superoxide levels [64,71,72]. Oxidative stress activates NF-κB through transduction pathways via PI3K and MAPK activation, which initiates apoptotic signal transduction and produces cell damage. In addition, oxidative stress reduces PPARγ activity and adiponectin levels. Both stimulate the AMPK protein, which is an inhibitor of NOX activity. Thus, oxidative stress inhibits this AMPK, and the action of NOX is promoted, which ultimately would further increase the release of NFkB [64].

At the cardiovascular level, the consequences of a spinal cord injury involve a significant morbidity and mortality impact. In general, plasma levels of catecholamines are usually low; however, there are situations in which loss of supraspinal control leads to a deregulated release of catecholamines. In addition, a hyperresponsiveness of alpha-adrenergic receptors is observed, which may be caused by both receptor hypersensitivity and a deficit of noradrenaline reuptake by the sympathetic neuron [73,74]. An altered lipid profile has also been observed in subjects with SCI, with elevated LDL and decreased HDL values. This increases the risk of heart disease and systemic atherosclerosis, which may be determined by low physical activity, inadequate diet and uncontrolled sympathetic activity [73].

Furthermore, the action of the autonomic nervous system on the promotion of inflammation may derive not only from the effects at the cardiovascular level, but also from its direct or indirect effect on the immune system. The specific mechanisms leading to immunosuppression in subjects with SCI are currently unknown, but different factors that may influence immune function have been observed. In a study conducted on laboratory rats, it was observed that the frequency of AD episodes increased as time since the injury passed, along with increased immunosuppression, probably due to the uncontrolled release of catecholamines and glucocorticoids [58]. Postganglionic noradrenergic fibers release catecholamines that target organs and lymphoid cells, causing a rapid increase of these cells in the blood. However, a chronic release of catecholamines, in the long term, produces the opposite effect, leading to leukopenia. Regarding the specific effect on the different types of immune response, the chronic increase of circulating catecholamines could have an inhibitory effect on the production of cytokines for the TH1 type immune response, but not so on the TH2 response [74,75]. It has also been observed that the relationship between elevated catecholamines and immune dysfunction occurs when the level of the lesion interrupts supraspinal control of the sympathetic innervation of the spleen, producing an increase of local catecholamines in this organ, even though the total circulating catecholamines in these subjects is lower, possibly due to an alteration of the innervation of the adrenal gland [56,75,76].

Likewise, just as immunosuppression has been described in subjects with spinal cord injury, there are also cases of long-term autoimmunity induced by spinal cord injury [77,78,79]. Thus, it has been described that spinal cord injury can expose CNS antigens to the circulation, which could provoke the activation of lymphocytes, which have specific receptors for these antigens. Activation of B cells can lead to the production of autoantibodies that ultimately lead to an autoimmune response and generate inflammation and neurotoxicity, resulting in poor recovery [79,80,81,82]. In an animal model, it was observed that rodents with B lymphocyte deficiency had a better motor recovery than control rodents, which suggests that there may be a relationship between these cells and the autoimmune response [83,84].

In a comparative study conducted by Saltzman et al. [85] on humans with and without SCI, the differential expression of a network of genes in peripheral blood was observed, to which a role in the structure and maturation of lymphoid tissue was attributed. Some of these genes were involved in inflammatory processes and in the regulation of B lymphocyte function. The importance of this network is that three molecules (BCMA, APRIL and BAFF) were encoded with a known role in producing autoimmune conditions [85,86]. BCMA is a receptor of the tumor necrosis factor (TNF) family that has been shown to mediate B cell survival and activation processes [85,87]. In addition, it is associated with an increase in the population of self-reactive cells [85,88,89,90]. Conversely, APRIL and BAFF are members of the TNF ligand superfamily, located in the germinal center of lymphoid organs, where they promote the survival, activation, maturation and differentiation of antibody-producing and memory B cells [85,91]. BAFF and APRIL can bind to BCMA and thus activate the NF-kB pathway, which triggers survival reactions for B cells [85]. Herman et al. [92] deepened the study previously carried out by Saltzman by increasing the number of people evaluated and confirmed changes in gene expression in subjects with spinal cord injury associated with lymphoid organs. In addition, it was observed that there was an increase in the expression of these genes as the lesion was higher than the T5 level.

**Figure 2:**
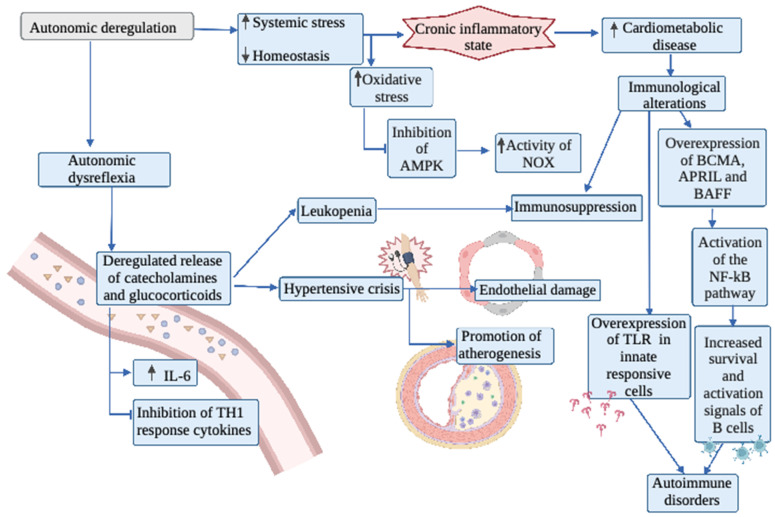
Overview of the role of inflammation and oxidative stress on the autonomic nervous system in patients with spinal cord injury. IL-6: Interleukin-6; AMPK: AMP-activated protein kinase; BCMA: B-cell maturation antigen; APRIL: A proliferation-inducing ligand; BAFF: B-cell–activating factor; NF-kB: Nuclear factor kappa B; TLR: Toll like receptors; NOX: NADPH oxidase.

Toll like receptors (TLRs) are innate pathogen recognition receptors present on immune cells that stimulate this process. TLR activation activates inflammatory mediators [93]. In the study conducted by Herman et al. [92], it was also observed that these TLRs were overexpressed in subjects with spinal cord injury above T5, which may promote an uncontrolled inflammatory response as has been studied in patients with sepsis who also present such TLR overexpression. TLR modulating agents are currently being studied in clinical trials for the management of autoimmune diseases and inflammatory responses and could be an alternative to be evaluated in subjects with SCI [92,94]. See the mechanisms overview in Figure 2.

## 4. Muscular System

Among the multiple consequences produced by SCI, immobility, disuse and muscular atrophy have an impact on the functioning of the locomotor system, which includes both bone tissue and skeletal muscle [95]. This deterioration is caused by both the denervation of the muscle resulting from acute spinal damage, and by the secondary damage resulting from the inflammatory reaction caused by neuronal death [96,97]. In addition, from the time of injury and in the long term, there is a decrease in blood flow to the regions below the lesion [98,99]. The muscle tissue in these patients shows a decrease in the radius of the fibers, loss of nuclei that alters regenerative capacity and a decreased number of mitochondria and their functionality [100,101]. In addition, within months, the change from type I fibers begins to occur until, within years, type IIx fibers predominate [17]. Associated with this process, increases in mediators that favor muscle protein degradation, such as myostastin [102], interleukin 6 [103,104] and TNF alpha [105,106] have been observed.

While the trophic effect of muscle innervation has been known since ancient times, the precise understanding of the mechanisms of chronic muscle involvement caused by SCI, resulting in continuous atrophy, possibly associated with oxidative damage, is still unclear [107]. Muscle atrophy is caused by an imbalance between proteolysis and protein synthesis. This process can be explained by muscle disuse, advanced age or exposure to microgravity conditions, resulting in loss of strength and a decrease in muscle mass [108,109,110]. In the study of this phenomenon, in an animal model, Kondo et al. [111] related oxidative damage to muscle atrophy caused by immobilization, observing an increase in thiobarbituric acid reactive substance (TBARS) and glutathione disulfide (GSSG) levels. Furthermore, in this study, a decrease in atrophy was found after vitamin E supplementation, thus attributing a large part of the origin of this phenomenon to oxidative stress. The main source of ROS production related to atrophy produced by immobilization has also been studied. Thus, Gram et al. [112] investigated how mitochondrial function and reactive oxygen species emission behaved in a group of young and older men subjected to two weeks of one-leg immobilization. First, it was observed that immobilization increased H_2_O_2_ production along with decreasing ATP formation by this organelle. Moreover, this effect on mitochondrial functioning was reversible after a six-week program of aerobic training on a cycloergometer [112]. Second, Liu et al. [113] suggest that the main source of ROS production associated with muscle atrophy is the NADPH oxidase 4 (NOX4), located in the sarcoplasmic reticulum. In addition, they investigated the role of calstabin 1 in this process, since it has been described that this protein favors the closure of the ryanodine receptor when muscle contraction ends; therefore, a dissociation between both proteins would decrease muscle strength. Thus, they designed a study in rats evaluating NOX4 expression after spinal cord injury at the T4 level. The results showed that an overexpression of NOX4 leads to an increase in the oxidation state, causing a prooxidant environment that dissociates calstabin 1 from the receptor of ryanodine 1, which could be one of the mechanisms of alteration of muscle contraction.

Hyperactivity of the renin-angiotensin system (RAS) is another phenomenon that has been widely studied, which is involved in several chronic pathologies, such as cardiac dysfunction. In this sense, Kadoguchi et al. [114] suggested that, if an increase in angiotensin II could produce systemic and cardiac altering effects, perhaps it could also have some implication on skeletal muscle function. Thus, in a study first carried out in an animal model, it was shown that rats administered with angiotensin II showed a greater loss of muscle mass and a decrease in the cross-sectional area of muscle fibers if compared to control rats. In addition, an increase in MuRF-1 and atrogyn-1, both part of the ubiquitin-proteosome degradative system, was observed [115]. In addition, although a decrease in the oxidative enzymatic activity of citrate synthase and mitochondrial complexes 1 and 3 was observed, superoxide generation from NADPH oxidase increased. Kadoguchi et al. [110] hypothesized that, if NOX2 is the main source of superoxide generation, and that there was an overexpression of a gene for NOX2 against muscle damage, angiotensin II could then play a role in NOX activity, thereby promoting the generation of muscle atrophy. To test their hypothesis, they studied an animal model in mice with a control group and a NOX2-deficient group, and angiotensin II was administered to both groups. Deletion of NOX2 prevented angiotensin II induced skeletal muscle atrophy by improving the balance between protein synthesis and degradation. In addition, they observed a decrease in the phosphorylation of AKT, which is a necessary signaling pathway in the process of protein synthesis. The authors noted that, therefore, NOX2 may be a therapeutic target for angiotensin II induced skeletal muscle atrophy.

**Figure 3:**
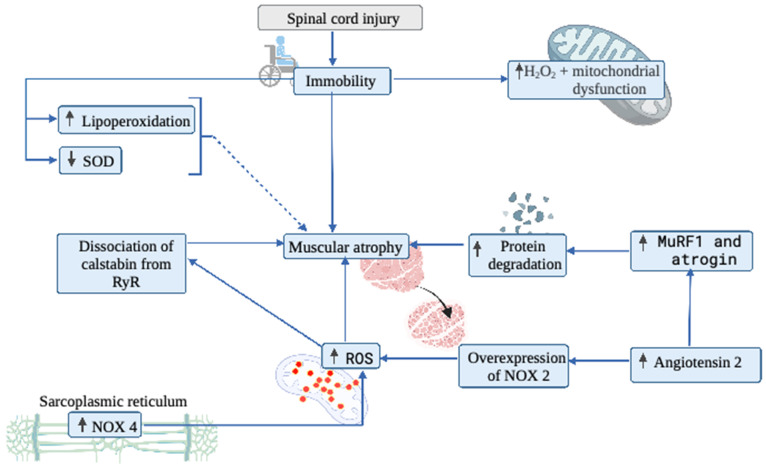
Overview of the role of inflammation and oxidative stress on muscle tissue in spinal cord injured patients. SOD: Superoxide dismutase; NOX4: NADPH oxidase 4; NOX2: NADPH oxidase 2; ROS: Reactive oxygen species; MuRF1: Muscle RING-finger protein-1.

In another area, Savikj et al. [116] conducted a study to assess whether spinal cord injury induces an oxidative imbalance leading to muscle damage. Regarding this study, they developed a comparative study in humans with complete spinal cord injury versus a healthy control group by obtaining muscle biopsies in the first, third and twelfth month after spinal cord injury. In this study, it was observed that in the skeletal muscle of spinal cord injured patients, there is an overexpression of xanthine oxidase in the first three months, and in a more chronic way at 12 months, increased levels of 4-HNE and a decrease in the content of superoxide dismutase 2 (SOD2). In addition, there is evidence linking oxidative stress with the activation of proteases at the skeletal muscle level, attributing to the mitochondria the main responsibility for the production of ROS in muscle atrophy [107,117,118]. Thus, Min et al. [107] evaluated the effect of mitochondrial ROS on muscle atrophy using a rat animal model focused on the administration of a mitochondrial antioxidant called SS-31, which is selective for the mitochondrial membrane. In this study, it was observed that immobilization compared to a control group of non-immobilized rats generated atrophy, increased ROS and the activation of proteolysis. When SS-31 was administered, ROS levels decreased, as did muscle atrophy and protein degradation [107]. To evaluate the effect of supplementation on muscle atrophy, Arc-Chagnaud et al. [119] conducted a study using a mixture of antioxidants and anti-inflammatory drugs (741 mg of polyphenols, 138 mg of vitamin E, 80 μg of selenium and 2.1 g of omega-3). Moreover, they observed the effect of physical deconditioning on muscle atrophy, using a 60-day head-down bed rest (HDBR) model in healthy volunteers. Out of the 20 subjects assessed by induced rest, only half received a mixture of antioxidants and anti-inflammatory drugs. The results showed that, after two months, all participants suffered loss of muscle mass. The authors highlighted the complexity of oxidative pathways that make it difficult to understand how oxidative stress influences muscle atrophy and questioned the effect of a nutritional intervention with antioxidants in preventing muscle deconditioning in long-duration space missions. However, it should be noted that the results of this study are in contrast with the results obtained by Min et al. [107] and Kondo et al. [111], who used antioxidants, which raises the question of how oxidative stress influences muscle atrophy. Figure 3 shows a summary of the mechanisms described in the paragraph above.

## 5. Conclusions

At the time a spinal cord injury is produced, a loss of motor capacity is evident, and a period of vital risk related to neurological compromise, instability of cardio-respiratory function and the development of infections is established. After this period, ranging from weeks to months, new relationships between different organs are established, now affecting functions that were initially unharmed. Thus, bone and muscle mass decreases, the respiratory system suffers restricted and inefficient ventilatory activity due to the affectation of its musculature, while the abnormal functioning of the autonomic nervous system will affect the action of various organs. In this review, we have described that the development of alterations in the redox state and the manifestation of increased inflammatory activity—that affects both individual tissues and at systemic level—is a common mechanism for the malfunctioning of these different but closely functionally integrated systems. The boundaries between the two processes are also blurred and one often promotes the emergence of the other. They are also involved in pathological processes as diverse as muscle atrophy and fibrosis secondary to disuse, as well as autoimmune diseases. Finally, it is interesting to note that, although much research is still pending (since there are many different events underlying these phenomena), application of antioxidant and anti-inflammatory therapies have been proposed either theoretically and through experimental studies [120,121,122,123] to delay or reverse the complications of this clinical condition. These lines of research offer a novel attractive approach that can have a significant impact on the quality of life of these patients.

## Data Availability

Not applicable.
